# Bayesian meta-analysis of the effectiveness of implementation science evidence in improving health outcomes for adolescent patients accessing ART in sub-Saharan Africa

**DOI:** 10.3389/fepid.2025.1547867

**Published:** 2025-09-17

**Authors:** Isaac Fwemba, Samuel Iddi, Thabane Lehane, Alfred Yawson, Jacques L. Tamuzi, Peter S. Nyasulu, Samuel Bosomprah

**Affiliations:** ^1^ Department of Biostatistics, School of Public Health, University of Ghana, Legon, Ghana; ^2^ Department of Epidemiology and Biostatistics, University of Zambia, Lusaka, Zambia; ^3^ Department of Health Research Methods, Evidence, and Impact, McMaster University, Hamilton, ON, Canada; ^4^ Division of Epidemiology and Biostatistics, Department of Global Health, Faculty of Medicine and Health Sciences, Stellenbosch University, Cape Town, South Africa

**Keywords:** Bayesian random-effects, meta-analysis, adolescent, retention, viral suppression, lost to follow-up, power prior, standard care

## Abstract

**Background:**

Implementation research (IR) studies and clinical trials have yielded conflicting results on improving treatment outcomes, especially among adolescent patients. To address this, we performed a Bayesian random-effects meta-analysis to evaluate the effectiveness of current IR interventions in improving retention in care and reducing viral suppression among HIV-infected adolescents.

**Method:**

A comprehensive search was conducted from 1 January 2000 to 31 December 2020. A Bayesian random-effects meta-analysis was performed using historical evidence from adolescent interventions conducted outside sub-Saharan Africa (SSA) and from adult-derived interventions within SSA. A Bayesian Copas random-effects model was used to account for publication bias and study variations. Power priors were used to weight the contribution of historical data to the analysis. Bayesian meta-analysis was particularly suited for this study since it allowed us to directly include prior assessments from several intervention studies into the pooled intervention data.

**Results:**

The pooled results from the 12 studies across eight African countries, involving 19,223 adolescent patients, showed significantly superior retention effects in adolescent-specialized interventions compared to standard care settings [odds ratio (OR) = 3.87; 95% credible interval (CrI): 0.94–10.82]. When 100% of data from eight observational studies on adolescent treatment outcomes were added to the analysis, the resulting OR was 3.02 (95% CrI: 1.01, 6.92). However, inclusion of 100% of historical data from adult randomised control trials (RCTs) reduced the retention effect to OR = 1.24 (95% CrI: 1.03–1.48). Regardless of whether adolescent historical data or adult RCT data were used, the associated posterior probability of benefiting from the intervention remained almost 1. There was no difference between standard care and specialized adolescent care in terms of virological suppression (OR = 1.27; 95% CrI: 0.57–2.32). However, specialized adolescent intervention achieved a superior overall retention rate of 59.7% compared to 52.1% under standard care.

**Conclusion:**

Current adolescent-specific interventions are effective in improving retention rates in HIV care. Evidence from adult interventions showed a reduced retention effect, suggesting that while adult-promising interventions may improve adolescent treatment outcomes, they may require modifications.

## Background

In recent times, there has been a resurgence of large-scale implementation research (IR) studies (1–3) focusing on human immunodeficiency virus (HIV) treatment outcomes in sub-Saharan Africa (SSA). The resurgence of these studies has been attributed to the fact that results from clinical trials may not yield the same results when implemented in real-world settings. Evidence from large clinical trial studies has shown that adults recruited into care, most of them above the age of 25 years, experience significant reductions in mortality and lost-to-follow-up rates, along with improvements in retention, compared to adolescent populations (4–6). However, evidence from observations and routine studies on improving treatment outcomes remains conflicting, and in practice, results have remained poor, especially among adolescents aged 15–19 years and youth above 20 years. Many countries have made significant progress in reaching the UNAIDS 95-95-95 targets for HIV epidemic control, with several countries already approaching or achieving them (7). However, children and adolescents living with HIV consistently fall behind adults in meeting these targets, with the third “95” target of sustained viral suppression remaining particularly challenging to achieve in these populations (7). Achieving the UNAIDS 95-95-95 targets by 2030 requires a strong focus on populations with suboptimal treatment outcomes, including adolescents living with HIV. While significant progress has been made in adult HIV care, adolescents continue to experience lower retention rates and poorer viral suppression, posing a major challenge to achieving the third “95” target. Understanding the effectiveness of implementation evidence-based interventions, such as educational, behavioral, and financial approached tailored to adolescents, is therefore critical for optimizing anti-retroviral treatment (ART) programs and ensuring that this vulnerable group fully benefits from global HIV treatment efforts.

Several implementation strategies and interventions have been implemented to improve treatment outcomes for this age group. In SSA, interventions such as adapting several models of care from adult populations to adolescent-specific settings (8), replacing clinical staff with community support groups (9, 10), and, in specific areas, modifying drug collection times to suit adolescent timings (11) have been proposed and are being implemented in selected countries.

Several challenges have been reported in related to these highlighted strategies, ranging from designing interventions that can address the complex health needs of adolescents living with HIV to finding studies quantifying the effectiveness of such interventions implemented in real-world settings (2). The few implementation research studies that are available have often been criticized for poor methodological rigor (12), consequently producing evidence that is difficult to generalize. Poor methodological rigor in most implementation research studies has been attributed to the lack of robust statistical methodologies capable of handling small sample sizes and to the presence of covariate and response measurement errors.

There is a need to use the available evidence to assess whether adapting the adult care model can lead to improved treatment outcomes for adolescent patients. There is also an urgent need to determine the effectiveness of the current interventions implemented in SSA settings. Such findings will help guide the modification of implementation strategies currently shaping and informing the implementation of treatment guidelines for HIV patients in care.

This study focused on retention and viral suppression because these HIV treatment outcomes are directly related to implementation outcomes, and there is no conclusive evidence regarding the effectiveness of IR interventions in improving treatment outcomes for adolescents living with HIV in care. We aim to provide a balance between what is known, particularly on the use of adult intervention to address treatment outcomes in adolescents living with HIV, and what is actually being implemented in the field regarding the effectiveness of current interventions. We believe that a Bayesian meta-analysis is appropriate for this study because it allows us to directly incorporate previous assessments from several other intervention studies into the pooled intervention data. Furthermore, Bayesian meta-analysis more clearly accounts for the degree of heterogeneity across studies, which is critical because utility values can be significantly varied among studies compared to existing meta-analyses of interventions aimed at improving retention in care and reducing viral suppression among adolescents living with HIV in SSA.

## Materials and methods

### Search strategies and selection criteria

EMBASE, MEDLINE, HINARI, JIAH, and Google Scholar were comprehensively searched for English-language studies published between 1 January 2000 and 31 December 2020 that evaluated viral suppression and retention of adolescent patients in care. Eligible studies were those conducted in sub-Saharan Africa and studied adolescent HIV patients. Specifically, the search strategy included terms such as “Adolescent interventions”, AND “sub Saharan Africa” OR “Viral suppression” OR “Retention” OR “Lost to Follow Up”, OR “Virological Failure”. We used both free text words and medical subject headings (MeSH) and a combination of the following words and their variations: adolescent, sub-Saharan Africa, retention, and viral suppression. Two investigators (IF and SI) examined and selected the studies, which were then uploaded to EndNote version ×9, and duplicates were removed. We also examined the reference lists of all included studies. The studies were not restricted to a specific study design. Studies that did not report on any of the predefined outcomes were excluded.

Two investigators (IF and SI) independently evaluated studies for inclusion. Any discrepancies or disagreements between them were resolved through discussion. When needed, a third reviewer (PN) was consulted. Studies were included if they met the following criteria:

• **Study population**: adolescents living with HIV, including adolescents (10–19 years), young adults (20–24 years), teens (10–14 years), and children (below 9 years).
• **Interventions**: included at least one implementation research strategy (intervention) aimed at improving either viral suppression or retention of adolescents in HIV care.
• **Comparator**: adult intervention studies comparing treatment outcomes with those of adolescents or health facility standard-of-care (SOC) models comparing outcomes with those from adolescent-specialized care models.
• **Primary outcome**: the study reported on retention and viral suppression as outcomes.

Two independent reviewers (IF and SI) extracted the data and resolved any differences by consensus. Absolute numbers were extracted; when odds ratios (OR) or risk ratios (RR) were reported, the authors recalculated the associated absolute numbers for the evaluated interventions.

### Statistical analysis

The study employed both frequentist and Bayesian approaches to analyze the meta-analytic data. Estimates obtained from the two approaches were compared. Both approaches reported measures of effects using ORs. For simple descriptive statistics, R software (version 3.5) and STATA software (version 14.1 SE; Stata Corporation, College Station, TX, USA) were used to calculate proportions and study totals. OpenBUGS, the open-source variant of WinBUGS (Bayesian inference Using Gibbs Sampling), was used to obtain posterior summaries via Bayesian hierarchical random-effects meta-analysis techniques. The outcomes of this review were reported according to the Preferred Reporting Items for Systematic Reviews and Meta-Analyses (PRISMA) guidelines (13).

A Bayesian random-effects meta-analysis was performed using historical evidence from adolescent data obtained from observational studies conducted outside SSA and from adolescent data from randomized studies conducted within SSA. However, since most implementation research studies are observational, and the historical studies included in this study were more observational rather than randomized, we adopted a power prior (14, 15) to control the influence of these observational studies on the estimated effects of the current study. The power prior distribution is a mechanism to estimate the effect of an intervention in a current study sample while accounting for the information from previous research. It offers a flexible approach for including different types of historical data into the prior distribution when analyzing newly collected data. The method is also used to evaluate the impact of external information on posterior summaries. The power prior is appropriate for evaluating the impact of subjective or informative priors on overall posterior estimates. Using the power approach in formulating informative prior information entails raising the likelihood of the pooled observation data to a power alpha (*α*). Setting *α* = 0 completely discounts the historical evidence, whereas using *α* = 1 incorporates 100% of the historical evidence from observational and randomized studies. In this study, we looked at various potential sources of heterogeneity. We focused on methodological and clinical differences among the studies included in the final analysis. For clinical heterogeneity, the team looked at the differences in patient populations and the details of the evaluated interventions, including the strategies used to deliver them. The outcomes measured across studies were also evaluated to ensure that only studies with similar outcomes, interventions, and populations were included. Similarly, subgroup analyses were conducted to evaluate the impact of different study designs on the overall estimates. This included exploring different data collection techniques and statistical methods used in the extracted studies. Thus, besides the random-effects meta-analysis, the study also employed subgroup analyses to examine the changes across different study subgroups to see whether heterogeneity could be explained by specific study characteristics. Similarly, meta-regression techniques were also used to investigate how study-level factors contribute to variation in effect sizes. Sensitivity analysis for this study was conducted using several values of *α* (0, 0.25, 0.5, 0.75, and 1.0). Their influence on the estimated effect size was monitored and subsequently reported. The choice of prior distribution was appropriately made to ensure the study aligned with a full Bayesian approach. This thinking motivated the construction of priors derived from different population groups. The use of adult data in combination with adolescent data reflects the clinical reasoning used by most clinicians, who often use effective adult interventions for adolescent populations. This conjecture was tested through the mechanism of combining the two population data sources. Similarly, the assumption of exchangeability was also explored to ensure that the parameter effects across the two groups were comparable. This was followed by a rigorous sensitivity analysis using the power prior mechanism. The power prior evaluated the effect of adult population data on the likelihood function constructed using adolescent data. In most cases, the posterior summaries were found to represent a compromise between the likelihood and the prior distribution. The effect of adding almost no information was compared with the effect of adding 10%, 20%, and 100% of the information from the constructed adult prior information. This therefore demonstrated that the estimates obtained were robust and not sensitive to the inclusion of external information within the modeling framework. The power prior mechanism has been recommended for evaluating the effect of informative priors on posterior summary estimates. Publication bias was assessed using the Bayesian Copas selection random-effects model (16) and Egger's asymmetry test (17). A funnel plot was used to assess the asymmetrical distribution of effect sizes.

The characteristics of the included studies also encompassed an assessment of the study quality.

**Figure 1:**
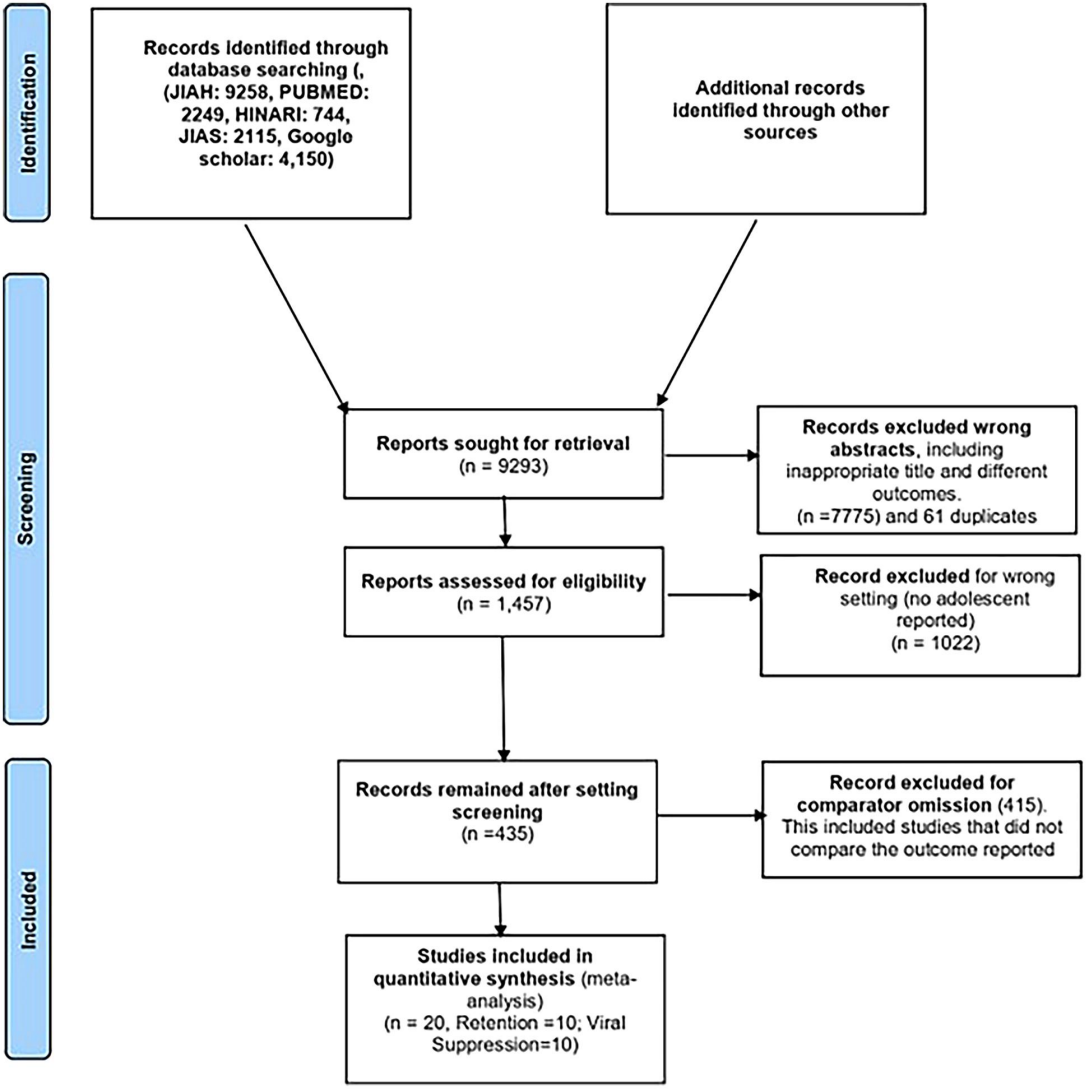
Flowchart of the PRISMA selection process and inclusion criteria.

The process of study identification and selection is shown in Figure 1. The literature search yielded 9,258 articles. After reviewing titles and abstracts, 176 studies were selected for review. Of these, 20 studies, comprising a population of 19,258 participants, were included in the meta-analysis. These studies were conducted across eight different sub-Saharan African countries, namely, Kenya, Malawi, Uganda, Rwanda, Mozambique, Zimbabwe, South Africa, and Tanzania. Two studies were multicenter cohort studies, while the remaining were single-country studies (9–11, 18–22). Three studies were excluded because they were duplicates of the other studies reviewed (18, 21). The detailed study selection process is shown in the flowchart in Figure 1. The commonly reported interventions included community-based interventions, teen clubs, adolescent clinics, youth red carpet programs (RCPs), youth- and adolescent-friendly clinics, community home-based care, and community-based support (CBS) for caregivers (Table 1).

**Table 1:** Study characteristics by interventions evaluated for retention outcomes.

No.	Reference	Period	Intervention type	Adolescent		Standard of care		Age
				Event	Total	Event	Total	
1	Ruria et al. (22)	2017	Adolescent and youth RCPs	424	430	234	430	15–19
2	Teasdale et al. (20)	2016	YAFS	37	274	71	576	10–24
3	Mackenzie et al. (11)	2017	Teen clubs	233	302	156	315	10–19
4	Fatti et al. (18)	2018	CBS	1,792	4,606	628	2,100	<16
5	Ferrand et al. (19)	2017	Community-based support for caregivers	27	155	30	165	6–15
6	Grimwood et al. (10)	2012	Community adherence	276	306	961	1,201	<16
7	Massavon et al. (23)	2014	Community home-based care	1,384	1,460	138	163	0–18
8	Zanoni et al. (21)	2017	Adolescent clinics	79	88	102	153	13–24
9	Amzel et al. (28)	2018	Community-based interventions	3,917	5,365	3,380	5,365	
10	Munyayi and van Wyk (41)	2020	Teen clubs	71	78	273	307	10–19

## Quality and risk of bias assessment

Of the 20 studies included, 8 were assessed using a systematic quality assessment tool (24) as good quality (10, 19, 25–28), seven were of moderate quality (9, 18, 21, 29), and the others were of low quality (9, 23, 30). Some common methodological problems noted included a lack of a sampling procedure, unclear data collection methodology, poor generalizability, and a lack of a theoretical perspective.

### Main study outcomes

## Main results

Two treatment-outcome meta-analyses were conducted using a Bayesian random-effects model. Posterior estimates and their associated credible intervals (CrIs) were obtained after incorporating adolescent and adult historical data. In Bayesian statistics, the prior probability refers to the probability of an event before new data are obtained. Both adult and adolescent historical data were used as sources of prior information and were combined with the main meta-analytic adolescent data using the power prior mechanism. Adolescent historical data were used purely as a source of prior information, while adult historical data were used to test the hypothesis that incorporating adult clinical information could provide estimates with the highest posterior probability of improving retention and reducing virological failure while in HIV care. Using adolescent historical data, the OR for retention was 1.73 (95% CrI: 0.44–3.97) and for viral suppression was 1.94 (95% CrI: 0.36–4.96), with associated standard deviations (SDs) of 0.98 and 1.03, respectively. Similarly, using the adult historical data, the OR for retention outcome was 1.2 (95% CrI: 0.001–1.39) and for viral suppression was estimated at 0.89 (95% CrI: 0.33–2.09), with the respective associated posterior standard deviations of 0.094 and 0.52, respectively (Table 2). This information was used to construct the prior distribution for the main meta-analytic data work reported in the following section.

**Table 2:** Study characteristics for viral suppression outcomes.

No	Author	Year	Intervention type	Intervention		Standard of care	
				Event	Total	Event	Total
1	Bermudez et al. (42)	2018	Economic strengthening	200	358	214	344
2	Mavhu et al. (43)	2020	Differentiated service	270	422	209	275
3	Amzel et al. (28)	2018	Community-based interventions	539	632	181	220
4	Fatti et al. (9)	2014	Community-based adherence support	3,876	5,908	3,279	5,908
5	Fatti et al. (18)	2018	Community-based support	1,705	2,100	2,893	4,606
6	Ferrand et al. (19)	2017	Community-based support for caregivers	44	86	63	94
7	Zanoni et al. (21)	2017	Adolescent clinics	178	196	157	196
8	Kabogo et al. (29)	2018	Universal testing and treatment policy	93	122	97	122
9	Nachega et al. (32)	2009	Community intervention	355	1,534	1,659	6,242
10	Nasuuna et al. (30)	2018	Intensified adherence counseling	62	274	15	72

### Retention in care

**Figure 2:**
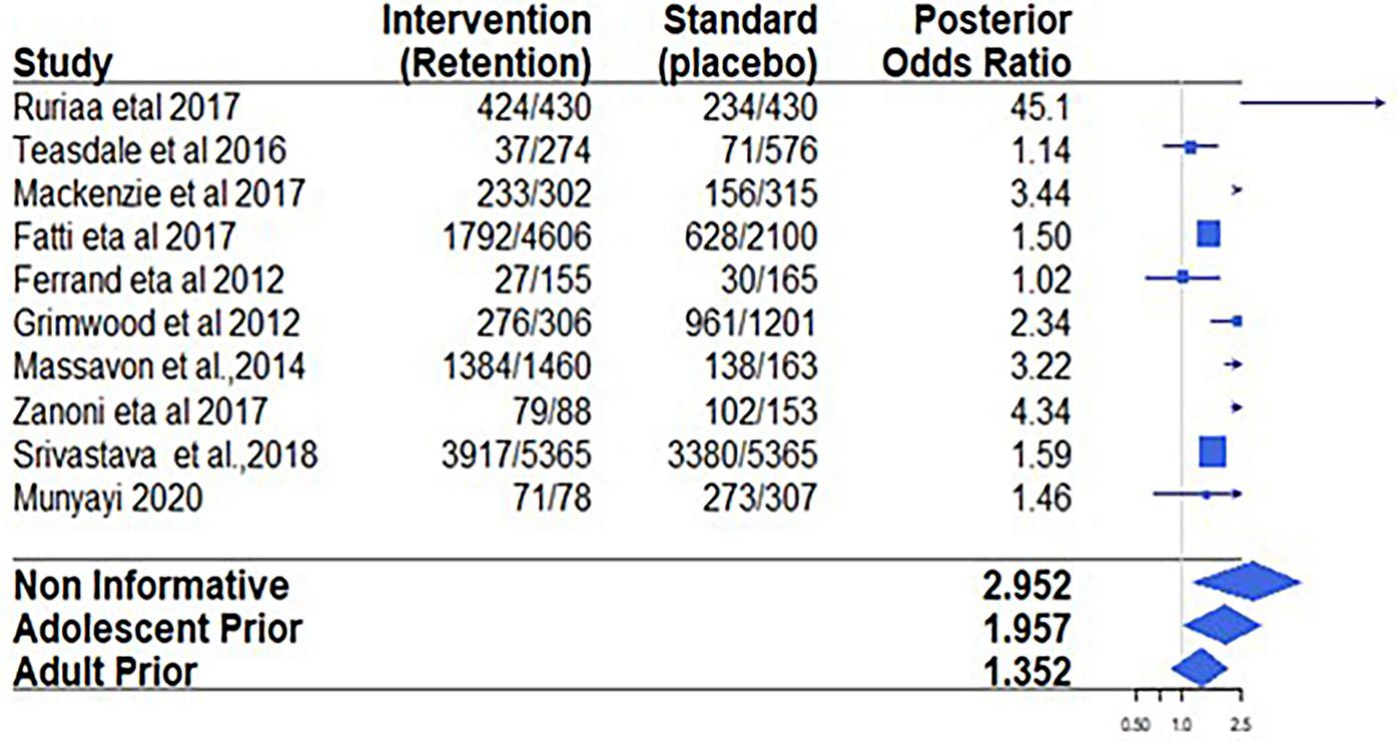
Forest plot of posterior probability of a retention benefit.

Eight studies reported retention as an outcome. Individual and pooled odds ratios for retention are shown in Table 3. Using a non-informative (vague) prior distribution to pool data from these studies, the retention odds ratio was 3.87 (95% CrI: 0.94–10.82), with an associated posterior probability of a retention benefit of 0.99. Among a total of 7,409 adolescents enrolled in adolescent-specific intervention programs, 4,537 were retained in care, compared to 2,541 retained out of 4,791 recruits in the standard-of-care (control) studies. This represents an increase in the absolute retention effect risk of 6.7%. Using the frequentist approach, similar results were obtained, with specific interventions showing better retention than standard-of-care interventions (OR = 2.90, 95% CrI: 1.57–5.36) (Figure 2).

**Table 3:** Pooled historical evidence for adolescent and adult RCT data.

Variable	Adolescent historical data				Adult historical data			
	OR	ln (OR)	SD	Precision	OR	ln (OR)	SD	Precision
Retention	1.73	0.54	0.98	1.04	1.2	0.18	0.094	113.17
Viral suppression	1.94	0.66	1.03	0.94	0.89	−0.12	0.52	3.7

Table 3 presents further results on retention outcomes using prior weights of 0.0, 0.5, and 1.0. We note that as the prior weight from the adolescent and adult historical evidence increased, the posterior mean of the retention effect and the posterior SDs for all parameters decreased, while the 95% credible interval became narrower. This reduction in interval size after including more information suggests that incorporating more prior information increases the confidence of the estimated effects. Most importantly, when *α* = 1 was used, implying using 100% of the historical evidence, none of the CrI values for the odds ratio coefficients contained 1.

Adding 50% of the historical evidence from adolescent studies resulted in a posterior odds ratio of 3.33, with the 95% credible interval narrowed to 0.98–8.98, which still includes 1. Similarly, adding 50% of historical evidence from adult studies resulted in a posterior odds ratio of 1.28, with a 95% credible interval of 0.98–1.65 and a posterior probability of 0. 95. Adding 100% of adult historical evidence reduced the estimated posterior effects to 1.24 (95% CrI: 1.03–1.48) (Table 4). Although with a wider interval, adding 100% of adolescent historical evidence also reduced the posterior OR to 3.07 (05% CrI; 1.01–6.92) (Table 4).

**Table 4:** Adjusted posterior odds ratio for adolescent improving retention outcomes with *α* representing power prior probabilities.

Estimates from adolescent-derived priors						Estimates from adult-derived priors				
*Α*	OR	2.50%	97.50%	Prob OR > 1	Between-study variation	OR	2.50%	97.50%	Prob of RR > 1	Between-study variance
0	**3** **.** **87**	0.94	10.82	0.99	1.868	**3**.**18**	0.96	10.73	0.94	2.16
0.5	**3**.**33**	0.98	8.98	0.99	1.863	**1**.**28**	0.98	1.65	0.96	2.70
1	**3**.**07**	1.01	6.92	0.99	1.861	**1**.**24**	1.03	1.48	0.99	2.74

Bold values are the posterior odds ratios that are Bayesian measures of effect.

With reference to the associated posterior probabilities of current IR interventions benefiting the adolescents living with HIV, i.e., the probability of obtaining an odds ratio of greater than 1, using adult RCT historical evidence increased this probability from .94 when 0% of evidence was included to 0.99 when 100% historical evidence was incorporated. Using observed evidence from the adolescent observational historical data, the probability of having an odds ratio greater than 1 remained consistently 0.99, regardless of the amount of evidence included.

### Reducing virological failure

**Figure 3:**
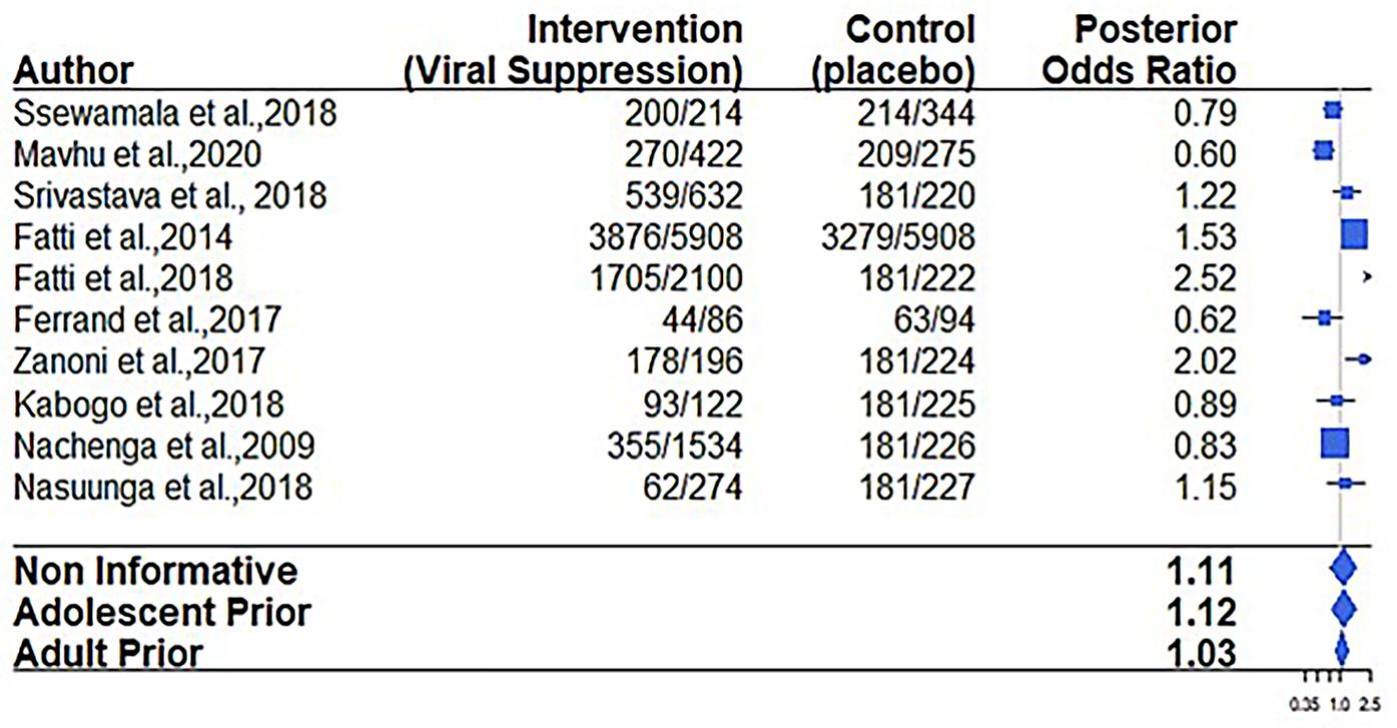
Forest plot of posterior probability of a viral suppression benefit.

Regarding the virological failure outcome, 6 out of the 12 papers considered for the meta-analysis reported on viral suppression (9, 19, 21, 31, 32). The overall virological failure rate score among adolescents receiving care in adolescent-oriented settings (interventions) employing various types of implementation strategies was 66.4% (1,141/1,719) compared to 56.1% (2,866/5,109) recorded in SOC settings. The results in Figure 3 and Table 5 indicate that the confidence interval includes 1, suggesting a non-statistically significant effect.

**Table 5:** Adjusted adolescent posterior odds ratio for adolescent virological failure with *α* representing power prior probabilities.

Viral suppression effect obtained after incorporating adolescent historical evidence from observational studies					Viral suppression effect obtained after incorporating adult historical evidence from randomized studies			
*α*	OR	95% CrI: 2.5–97.5	Probability of RR > 1	Between-study variation	OR	95% CrI: 2.5, 97.5	Probability of RR > 1	Between-study variation
0	1.84	0.48–4.86	0.6712	3.226	1.86	0.01–4.95	0.81	1.93
0.5	1.40	0.53–2.78	0.673	3.273	1.75	0.00–1.56	0.83	1.79
1	1.27	0.57–2.32	0.6716	3.235	1.72	0.00–1.53	0.85	1.72

The posterior probability of benefiting virologically when using adolescent historical evidence (0.67) was found to be consistently lower than when using adult historical evidence (0.85).

### Constructing prior information and assessing impact on the posterior distribution

Using a “non-informative” prior distribution to pool data from observational studies, the retention odds ratio was 2.95 (95% CrI: 1.44–6.26), with a posterior probability of a retention benefit of 1 (Table 6). Using an “informative” prior distribution, the eight adolescent observation retention studies were pooled to obtain an OR of 1.63 (95% CrI: 0.50–4.08). When adolescent data from SSA were added, the OR was 1.67 (95% CrI: 1.03–3.36), with a posterior probability of a retention benefit of 1.0. The posterior probability measure was used to determine the probability of obtaining an OR value above 1.0, which was a probability of benefiting from the intervention.

**Table 6:** Effects of adolescent and adult historical data on adolescent intervention estimates.

Assumptions	Prior distribution	Odds ratio (retention)	Odds ratio (viral suppression)	Probability of retention OR > 1	Probability of viral suppression OR > 1
External Information	Non-informative prior distribution	2.95 (1.44–6.26)	1.11 (0.73–1.60)	1.0	1.0
Synthesis of observation and randomized studies	Informative prior distribution—adolescent interventions	1.96 (1.03–3.36)	1.12 (0.75–1.61)	1.0	0.71
	Informative prior distribution—adult interventions	1.23 (0.96–1.55)	1.04 (0.88–1.24)	1.0	0.66

Using the adult retention informative prior distribution, 10 adult studies from SSA were pooled to obtain a non-significant OR of 1.40 (95% CrI: 0.98–1.45) (Table 6). When combined with adolescent data from SSA, the estimate OR was 1.12 (95% CrI: 0.75–1.61), with a posterior probability of a retention benefit of 0.71, which remained non-significant (Table 6). However, the use of non-informative and informative prior distributions for the viral suppression outcome did not improve the intervention estimates.

### Adolescent

 The Bayesian estimates obtained from eight observation studies reporting retention outcomes: OR = 1.63 (95% CrI: 0.50–4.08); ln(OR) = 0.49; SD of OR = 1.19, variance = 1.416, and precision = 0.71.
 The Bayesian estimates obtained from six adolescent observation studies reporting viral suppression outcomes: OR = 1.72 (95% CrI: 0.57–4.03); ln(OR) = 0.54; SD of OR = 1.16, variance = 1.42, and precision = 0.704.

### Adult

 The Bayesian estimates obtained from 10 studies reporting retention outcomes from SSA: OR = 1.40 (95% CrI: 0.98–1.45); ln(OR) = 0.337; SD of OR = 0.09, variance=0.25, and precision = 15.87.
 The Bayesian estimates obtained from 10 studies reporting viral suppression outcomes: OR = 1.02 (95% CrI: 0.84–1.22); ln(OR) = 0.0227, SD of OR = 0.10, variance = 0.09, and precision = 112.31.

### Publication bias assessment and sensitivity analysis

Overall, the Copas selection model showed that there was no publication bias affecting the posterior mean effect size estimates. The estimated publication bias for studies reporting retention outcomes was 0.071. A similar but smaller correlation coefficient was reported for viral suppression. This finding was confirmed by the frequentist Egger test (*b* = 0.64, *t* = 3.13, *p* = 0.322), which revealed that small studies did not bias the result toward positive outcomes. We conducted a sensitivity analysis by excluding studies that were conducted after 2014, not implemented in community settings, or included youth HIV patients. The results from these sensitivity analyses did not change much from those obtained from the complete analysis reported in the main section.

## Discussion

This meta-analysis used a Bayesian approach to synthesize the available few high-quality studies to determine whether the current treatment-outcome interventions implemented in real-world settings are sufficiently effective in improving retention and viral suppression among adolescent HIV patients in care.

We applied a rigorous statistical approach to analyze meta-analytic data from different study populations. The use of the Copas selection model to account for publication bias and between-study heterogeneity, together with the use of the Bayesian random-effects model as the main pooling strategy, illustrates the Bayesian modeling innovations employed in this work. Using these techniques, the study established comparable estimates of adolescent retention rates in care across SSA. Pooling evidence from 12 studies conducted across more than 8 countries demonstrated that the current IR studies implemented in SSA are 59.7% more effective in improving retention rates in care than standard-of-care interventions. Using adolescent and adult historical evidence also showed that IR interventions were 3.0 and 1.28 times more effective, respectively, than standard-of-care models in retaining adolescent patients in care. Compared to a systematic review of 10 studies evaluating interventions to improve retention in care among adolescents living with HIV in SSA, the overall number of studies for each type of intervention remained small, the quality of evidence was inconsistent, and—even for interventions with more available evidence—the findings were inconclusive (33). Another systematic review including 30 studies accessing psychosocial interventions for improving engagement in care among adolescents and young people living with HIV in the United States, SSA, and Southeast Asia found no significant impact of psychosocial interventions on retention in care (34). The effectiveness of adolescent-specific interventions for individuals living with HIV, implemented in real-world settings, over standard-of-care settings in retaining patients in care has also been reported in several other contexts (35–37). However, it is worth noting that the reported effectiveness of these interventions remains lower than that observed among adults in the same region, where retention rates average between 65% and 70% (38). This finding is also lower than those of two studies conducted in South Africa, which reported adolescent retention rates exceeding 83% (35) and 89% (21). The higher retention rate reported in a South African study might have been influenced by a number of factors, including variability in age definitions and the use of programmatic data, which could have inflated the estimates. Our findings revealed that using a non-informative vague prior distribution to pool data resulted in an odds ratio of 3.87 (95% CrI: 0.94–10.82) for retention to care, with a posterior probability of a retention benefit of 0.99. Although clinically meaningful, these findings should be interpreted cautiously given their statistical insignificance. Similarly, with a broader interval, adding 100% of adolescent historical evidence reduced the posterior odds ratio. Even while these findings should be interpreted in terms of clinical significance, that is, they are substantial enough to have practical meaning or impact in real-world settings, the lower bound of 1.01 indicates borderline statistical significance.

Surprisingly, our study found no significant effect on viral suppression. Using all *α* values to control the weight of prior information from adolescent historical observational studies (RR = 1.84–1.27) and the adult RCT historical evidence (RR = 1.86–1.72) did not improve the study estimates. The reported results suggest that the current interventions for adolescents living with HIV, as implemented in various settings, may not produce outcomes different from those recorded under standard pediatric care. Various studies highlighted no improvement in viral suppression in real-world interventions (35, 36). Similarly, two systematic reviews showed that such interventions had no significant impact on improving viral load suppression in adolescents and young people living with HIV in SSA, the United States, and Southeast Asia (33, 34).

However, the number of adolescents living with HIV presenting with a better viral suppression rate was higher in adolescent-specific interventions (66.4%) compared to the standard-of-care models (56.0%). The estimated viral suppression rate falls within the reported range for Africa, which varies between 27% and 89% (39).

Using the Bayesian approach, this study was able to determine the level of uncertainty surrounding the conclusion on the effect of IR HIV treatment. The change in the level of uncertainty was assessed using the power prior technique, which also helped control the amount of historical information influencing the posterior estimates in the modeling process. Controlling the amount of evidence being included in the modeling framework is appreciated, especially in studies like ours, where heterogeneity exists between historical evidence and the current study—due to differences in sample sizes and the complex of interventions that vary across studies. From the results presented in the aforementioned table, it can be seen that the inclusion of historical data (14, 15) influenced the posterior estimates for the two reported outcomes. However, the analysis has also shown that evidence derived from adult-oriented settings is not a magic bullet for improving treatment outcomes in adolescent patients, especially regarding viral suppression. As noted through the use of the power prior and Bayesian mechanism, youth and adolescents living with HIV still exhibited lower effects of retention in care and showed no significant improvement in achieving better and sustainable viral suppression (40) compared to standard pediatric care.

When the main adolescent meta-analytic data were pooled using a 0.0 probability of including the observed evidence from adolescent historical observational studies, the posterior probability that the adolescent intervention provides a retention benefit, i.e., RR > 1, was 0.99. When an informative prior was constructed with a 1.0 probability of including the observed evidence, the posterior probability of a retention benefit was still 0.99. When adult historical evidence from RCTs was used with a 1.0 probability of inclusion, the posterior probability of a retention benefit remained close to 1 (0.99), despite reducing the posterior odds ratio to 1.24.

However, the viral suppression outcome produced a different posterior probability of benefit for adolescents in care. When a 0.0 probability of evidence was incorporated into the main meta-analytic data from adolescent observational historical studies, the posterior probability of a viral suppression benefit given the data was 0.67. Using a 1.0 probability of observed evidence from the same data, which corresponds to using 100% of the evidence, still resulted in a 0.67 posterior probability of benefiting virologically from the current interventions in SSA. Interestingly, given the observed data, using a 0.0 probability of historical evidence from adult RCTs to combine with the meta-analytical data resulted in a 0.81 posterior probability of benefiting virologically. When 100% of the observed evidence was used, the posterior probability of adolescents benefiting virologically from IR adolescent interventions increased to 0.85. The sensitivity of the posterior probability to the inclusion of adult RCT historical evidence suggests a lack of robustness in the prior range of plausible values. The difference in effect sizes from the two historical metadata sources represents a lack of convergence between the information drawn from adult-oriented models and adolescent-oriented interventions. This difference reveals the need to modify current treatment guidelines and strategies to improve adolescent treatment outcomes. However, the robustness of the hyperparameters exhibited in the historical evidence from adolescent observational studies demonstrates the convergence of evidence from different sources on different treatment outcomes for adolescent patients in care. Convergence is also evident in both the posterior probabilities of benefit and the posterior odds ratio effects, which did not change much within each treatment outcome, regardless of the inclusion of evidence. The differences noted in the adult RCTs, however, are a clear demonstration of how reliance on current adult data in clinical practice can overestimate the probability of benefit while producing marginally lower effect sizes than those observed in observational studies implemented in real-world settings. The results also indicate that adult innovations promising better treatment outcomes may not necessarily translate into improved outcomes for adolescent patients. This observation is also consistent with findings from most implementation strategies evaluated in the reported intervention studies. The characteristics of these interventions included, at most, complex interventions such as community-based support, community adherence programs, and community-based caregiver support, all anchored and relevant to the social context and appealing to the specific health needs of adolescents living with HIV in the region. The studies extracted also reported adolescent-specific interventions, such as youth- and adolescent-friendly services (YAFS), dedicated adolescent clinics, and teen clubs. The implementation of complex interventions addresses the complex nature of the health needs of adolescents, who may require rather more complex interventions than those implemented in adult care settings. This observation may explain why most clinical assumptions about adolescent treatment outcomes, especially for HIV interventions, yield different results when implemented in real-life settings. Our results further suggest that evidence from adult-specific interventions that are promising for improving HIV treatment outcomes needs to be interpreted in light of the above-mentioned evidence.

There are a few limitations to our study findings. First, the number of studies included was very small. Lack of credible studies may have affected the statistical power of our estimates. Second, the study used meta-analytical historical data for adolescent patients from other parts of the world with different settings, cultural understandings, and practices. It is possible that estimates derived from populations outside of SSA may exhibit different health-seeking behavior from those within SSA. Thus, using information from populations outside of SSA may be less informative for our study outcomes. However, incorporating historical data from other regions of the world provided the benefit of using credible evidence with higher statistical power to inform the analysis of the current data. Third, the use of adult historical data from RCTs could be contentious, as these interventions were conducted in populations that differ from those currently being studied. There are several reasons however to believe that adult populations and adolescents in HIV care from SSA could be considered commensurate, as they are managed within the same treatment environments, cared for by the same health practitioners, and exposed to the same healthcare systems. Further, the studies included in this meta-analysis reported variable age definitions, which were not homogeneous. This variation in adolescent definitions likely reflects the lack of standardized definitions for the adolescent population. This calls for studies with standardized definitions so that future findings can be generalized in SSA. Misclassification of children and young adults into the adolescent group may have resulted in either underestimation or overestimation of odds ratios in this study. Furthermore, the considerable uncertainty in some results may be due to heterogeneity between studies. Given the above limitations, these study findings must be interpreted with caution. While this study also acknowledges the notable impact of pharmacologic advances in ART, including the transition from nevirapine to efavirenz, protease inhibitors, and more recently dolutegravir, as well as the shift from twice-daily to once-daily dosing regimens, these changes have significantly contributed to improved tolerability, adherence, and viral suppression. However, our findings indicate that the effectiveness of these efficacious treatments among adolescents is also strongly influenced by the implementation strategy or delivery mechanism used. In this context, such strategies may have a mediating or moderating effect on treatment outcomes. This underscores the importance of not only using effective treatment regimens but also adopting context-appropriate, responsive, and adolescent-centered delivery strategies to achieve optimal outcomes in HIV treatment and care. While we acknowledge the significant effect sizes noted in this study, we also feel that accounting for changes in improvement in treatment protocols and guidelines could have positively impacted the effectiveness of these interventions. It is noted that the incremental improvements in the standard of care over the years might have resulted in more effectiveness of these interventions.

The study has successfully pooled data from eight African countries. The effectiveness of adolescent-based interventions implemented in the SSA context is evaluated for the first time. What is already known is based on estimates from programmatic data and a few meta-analyses studied that are either based on single-African-country studies or studies conducted outside SSA. Testing the hypothesis that promising adult evidence can optimize treatment outcomes for adolescents living with HIV care is also another innovation and broader contribution of this study.

## Conclusions

The evidence from this study supports the notion that current implementation research interventions aimed at improving general treatment outcomes for adolescent patients are effective. The results of this study also suggest that interventions such as community-based support groups, community adherence groups, teen clubs, and youth- and adolescent-friendly clinics, as well as evaluated policy modifications, were associated with superior treatment outcomes compared to those recorded in standard-of-care settings. However, it is worth mentioning that, even though current interventions are effective in improving retention in care for adolescents living with HIV, they are not as effective as adult interventions implemented in the same clinical and social setting. Using adult evidence to inform treatment guidelines for adolescent patients overestimates the true probability of benefit drawn from these promising adult interventions. Thus, based on the evidence from this study, we can argue that the most promising adult interventions implemented in an adolescent-oriented care setting or adult settings targeting adolescent patients need modification and adaptation to address the special needs of adolescent patients.

In this study, we observed that adolescents living with HIV in care need more complex interventions compared to adults. We, however, recommend that another study using network meta-analysis would be helpful to provide head-to-head comparisons of intervention effectiveness, particularly in light of different population groups that have been misclassified in adolescent definitions.
